# From digital access to social connectedness: the digital divide, bonding social capital, and depressive symptoms among older adults in China

**DOI:** 10.3389/fpsyt.2026.1845066

**Published:** 2026-06-16

**Authors:** Yan Wang, Luwen Zhang, Wenbin Wang

**Affiliations:** 1 School of Humanities, Jilin Agricultural University, Changchun, China; 2 School of Psychology and sociology, Jilin University, Changchun, China

**Keywords:** bonding social capital, depressive symptoms, digital divide, healthy aging, older adults

## Abstract

**Introduction:**

As population aging and digital transformation continue simultaneously in China, the digital divide among older adults has become an increasingly important social issue. This study examines the associations between multiple dimensions of the digital divide and depressive symptoms among older adults, as well as the potential role of bonding social capital.

**Methods:**

Drawing on three waves of data from the China Family Panel Studies (CFPS, 2018–2022), this study employs two-way fixed effects models and mediation analyses to examine the relationships between digital access, digital usage, digital outcomes, and depressive symptoms among older adults. Robustness checks were further conducted using propensity score matching (PSM), sample restriction adjustments, and replacement of the dependent variable.

**Results:**

Internet access was significantly associated with lower levels of depressive symptoms among older adults (p < 0.05). Compared with non-Internet users, entertainment-oriented, instrument-oriented, and mixed Internet use were all significantly associated with lower depressive symptoms (all p < 0.05). Digital outcomes were also negatively associated with depressive symptoms (p < 0.01). Bonding social capital showed significant indirect pathways linking all dimensions of the digital divide and depressive symptoms, with mediating proportions ranging from 5.95% to 26.67%. Period heterogeneity analyses further indicated that the associations remained generally stable before and during the COVID-19 period, although mixed Internet use exhibited a significant structural difference across periods (p = 0.036).

**Discussion:**

The findings suggest that the digital divide is closely associated with the mental well-being of older adults, while bonding social capital constitutes an important social pathway linking digital engagement and psychological health. Policy efforts should move beyond technological access toward broader digital empowerment and the construction of a more inclusive digital society for aging populations.

## 1 Introduction

Chinese society is currently at the intersection of accelerated population aging and deepening digital transformation, and the interaction of these two processes is reshaping both social operations and individual life structures. As the Internet becomes deeply embedded across various domains of society, digital technology has evolved from a tool for information dissemination into a fundamental structural force, which connects individuals, organizations, and society. among However, disparities in access conditions, usage capabilities, and application patterns of digital technologies have gradually transformed the digital divide into a new form of social inequality in contemporary society (1, 2). Older adults, in particular, are more likely to be disadvantaged in the process of digital inclusion due to physiological decline, cognitive barriers, and changes in social roles (3). The increasing intersection between digitalization and population aging has not only created structural barriers for older adults in accessing everyday public services, but has also heightened their risk of weakened social connections. Ultimately, such social marginalization resulting from digital exclusion may substantially increase the likelihood of depressive symptoms.

Depressive symptoms, as one of the most common negative psychological states among older adults, have become an important dimension for assessing their psychological well-being and align with the World Health Organization’s definition of mental health (4). Existing studies suggest that the digital divide can influence depressive symptoms through multiple pathways. On the one hand, the diffusion of digital technologies facilitates access to information and the expansion of social networks, enabling individuals to obtain greater emotional support and social resources, which may help alleviate depressive symptoms to some extent (5). On the other hand, some studies indicate that digital use may lead to issues such as information overload, Internet addiction, and social comparison, thereby exacerbating negative emotional experiences (6, 7). These findings suggest that the relationship between the digital divide and depressive symptoms is complex. The underlying mechanisms still await further investigation.

In recent years, a growing body of research has examined the heterogeneous associations between the digital divide and depressive symptoms from the perspective of social relationships. Among the various explanatory perspectives, social capital has been regarded as an important meso-level variable linking individuals, social relationships, and institutional environments, exerting substantial influence on individual well-being through interactions within social networks (8). In the field of aging research, abundant social capital can alleviate depressive symptoms by providing emotional support, increasing access to material resources, and promoting social participation (2). In the context of a digital society, digital technologies have transformed patterns of social interaction and may facilitate the strengthening and expansion of social networks (9), thereby further influencing depressive symptoms among older adults.

However, several limitations remain in the existing literature. First, most studies primarily focus on Internet access or frequency of use, while relatively few have systematically examined the issue from the multidimensional perspectives of digital access, digital usage, and digital outcomes. Second, prior research has paid greater attention to the direct effects of digital technologies on mental health, with insufficient consideration of the underlying social mechanisms. Third, existing studies have largely adopted a static perspective and have paid limited attention to the dynamic changes in the relationship between the digital divide and depressive symptoms among older adults amid rapid digital development.

This study contributes to the literature in three main aspects. First, grounded in digital divide theory, this study systematically examines the effects of digital technologies on depressive symptoms among older adults from the three dimensions of digital access, digital usage, and digital outcomes. Second, this study further incorporates perspectives from social networks and social capital to explore the social mechanisms through which digital technologies influence mental health among older adults. Third, using three waves of CFPS data, this study further investigates whether the effects of the digital divide have undergone dynamic changes in the context of digital development and the COVID-19 pandemic.

Building on this gap, this study draws on data from the 2018, 2020, and 2022 waves of the China Family Panel Studies (CFPS) to systematically examine the associations between the digital divide and depressive symptoms among older adults, as well as the underlying social mechanisms. It aims to systematically examine how digital divide are associated with depressive symptoms among older adults, as well as their potential pathways and temporal dynamics. Specifically, this study addresses three research questions. First, whether digital access, digital usage, and digital outcomes are associated with lower levels of depressive symptoms among older adults. Second, whether these associations remain stable during periods of rapid digital transformation in China. Third, whether social capital functions as a mediating pathway linking the digital divide to depressive symptoms. To address these questions, the following sections review relevant literature, synthesize theoretical debates and empirical findings, and conduct further analysis.

## 2 Literature review and hypotheses

### 2.1 The evolution of digital divide theory

Since the 1990s, digital divide theory has evolved from a single-dimensional focus on access disparities to a multidimensional framework. Early studies concentrated on inequalities in device ownership and Internet connectivity, referred to as the “first-level digital divide” (10). At this stage, the digital divide was largely conceptualized as a binary distinction of “connected” versus “not connected.” Some scholars assumed that Internet access would automatically translate into social benefits derived from digital technologies. However, with the widespread diffusion of the Internet, researchers increasingly argued that access alone does not guarantee meaningful benefits. Physical access does not necessarily imply substantive digital inclusion (11). Hargittai (13) proposed distinguishing access inequality from skill inequality, marking the emergence of the “second-level digital divide”. This perspective highlights heterogeneity in technical skills, digital literacy, and usage preferences. Subsequent studies further differentiated this level into technical competence and informational literacy (13, 14). This stage represents a shift away from technological determinism toward an emphasis on how usage patterns shape the conversion of digital resources. As digital technologies increasingly permeate social life, scholars recognized that even with access and skills, individuals may not achieve equivalent benefits. This gave rise to the “third-level digital divide,” which focuses on disparities in digital outcomes (6). This dimension concerns whether digital usage translates into tangible outcomes such as education, employment, or psychological well-being (15, 16). Van Dijk’s (17) resource appropriation theory integrates these levels into a dynamic process of resource conversion. From this perspective, the digital divide is no longer limited to access inequality. It is understood as a multidimensional structure comprising digital access, digital usage, and digital outcomes. It may also reinforce broader patterns of social inequality.

Within this framework, older adults have become a key focus in digital divide research. Age is a strong predictor of digital access and is closely associated with digital skills and digital outcomes (17). Even after overcoming barriers to digital access, older adults often face substantial challenges in digital usage and digital outcomes. Their disadvantages are not only from limited access but also from skill deficits and cognitive constraints. These factors restrict their ability to translate digital engagement into tangible benefits. Therefore, examining the associations between digital access, digital usage, digital outcomes, and depressive symptoms among older adults is both theoretically and practically significant. Moreover, as digitalization continues to advance in China, the role of the digital technology has evolved. It has shifted from a tool of initial exposure to an integral part of daily life. As a result, the effects of the digital divide may not be static. The associations between its three dimensions and depressive symptoms may exhibit dynamic patterns over time. This potential temporal variation provides an important context for understanding long-term associations.

### 2.2 Digital divide and depressive symptoms among older adults

Research findings on the relationship between the digital divide and depressive symptoms among older adults are mixed. Overall, digital usage is often associated with lower levels of depressive symptoms. By overcoming barriers in digital access, the Internet offers new channels for social interaction. Frequent communication via social media can increase social contact and perceived support, thereby reducing loneliness and depressive risk (18, 19). In addition, the Internet serves a compensatory function in information access. Compared with traditional channels, it enables more efficient access to health, medical, and social service information (20, 21). This may alleviate inequalities in access to health resources. Furthermore, the development of digital services such as telemedicine helps address uneven resource distribution and urban–rural disparities (Daniels et al., 2024). These services are associated with reduced depressive risk and suicidal ideation among older adults (22). Thus, digital access and basic technological resources may empower older adults and are associated with lower levels of depressive symptoms.

However, the positive effects of digital technologies vary across contexts. These variations are largely shaped by differences in digital usage and digital outcomes. Some studies find that excessive or problematic social media use is associated with increased anxiety and depressive symptoms (23, 24). When digital environments serve as a form of escape from real-life stress, they may intensify social isolation (25). This can disrupt normal socialization processes and increase depressive risk. In addition, risks such as online fraud, information overload, and social comparison may generate negative emotional responses among older adults (26). Notably, the digital divide may reinforce the “inverse care law”. Vulnerable groups who need digital health services the most may be excluded due to access barriers or lack of skills (27). As a result, they may exhibit higher levels of depressive symptoms compared to peers. Such low-return digital engagement may further widen intra- and intergenerational health disparities (6, 28). Therefore, the relationship between digital technologies and depressive symptoms depends on specific usage patterns and realized outcomes.

Overall, a multidimensional approach is therefore necessary to examine these associations. Based on the above discussion, this study proposes the following hypotheses.

Hypothesis 1a: Digital access is associated with lower levels of depressive symptoms among older adults.

Hypothesis 1b: Entertainment-oriented, instrument-oriented, and mixed digital usage are associated with lower levels of depressive symptoms among older adults.

Hypothesis 1c: Digital outcomes are associated with lower levels of depressive symptoms among older adults.

### 2.3 Explanatory pathways of social capital in the association between the digital divide and depressive symptoms

The relationship between the digital divide and depressive symptoms among older adults is inherently complex, and focusing solely on the direct association between the two remains insufficient for explaining the underlying connections. It is therefore necessary to examine the underlying social mechanisms. Existing studies suggest that the Internet is not a direct determinant of psychological outcomes. Its effect on depression largely depends on whether individuals can translate Internet use into tangible resources and support in real life. Digital technologies may reshape the structure of individuals’ embedded social networks and interaction patterns, thereby influencing their capacity to obtain social resources and support (29, 30). In this process, social capital, as an important resource embedded within social relationship networks, may constitute a crucial pathway linking the digital divide to depressive symptoms.

Social capital refers to the resources that individuals and groups can access through formal and informal social networks. Its core components include social networks, social norms, civic engagement, and trust, all of which facilitate coordination and cooperation for mutual benefit (31). In terms of classification, Putnam distinguishes between bonding social capital (BSC) and bridging social capital (BrSC) based on the strength and structural characteristics of social ties. BSC primarily exists within close-knit networks such as family members, friends, and neighbors, emphasizing emotional support and mutual aid. In contrast, BrSC is embedded in broader, weaker, and more heterogeneous networks, facilitating information flow and resource acquisition (31).

Previous studies have suggested that digital technologies may exhibit distinct mechanisms of association with different forms of social capital. The Internet may not only strengthen existing strong-tie networks, but also facilitate the expansion of intergroup connections (12). Meanwhile, sociomotional selectivity theory posits that, with aging, declining physical functioning, and withdrawal from occupational roles, their social networks gradually contract (32, 33). Cross-group interactions associated with bridging ties diminish, while reliance on strong ties, such as family and close friends, intensifies (34). Under such conditions, BSC becomes the primary source of emotional support and daily care in later life, playing a critical role in mitigating the risk of depression.

In the digital era, digital access and usage provide new opportunities for the reconstruction of BSC among older adults. Through social media, they can maintain frequent interactions with their children, relatives or friends, overcoming spatial and temporal constraints. This form of online co-presence can compensate for disruptions in face-to-face contact caused by geographic distance, thereby alleviating social isolation resulting from reduced offline interactions (35). In addition, the Internet may generate a social reinforcement effect. Empirical evidence indicates that online interactions can strengthen trust and perceived reciprocity among existing network members (19). However, the relationship between digital technologies and social capital remains contested in the existing literature. Some studies argue that online interactions may substitute for offline engagement, potentially weakening real-world social networks (36). Moreover, the spread of negative information and the uncertainties associated with online anonymity may undermine trust in social relationships. Consequently, the association between the digital divide and social capital is highly complex, and the direction of its effects still requires further empirical examination.

Some research demonstrates that social capital is a crucial social resource shaping individual psychological well-being (37). Through social networks, individuals gain emotional support, access to information, and social recognition, all of which contribute to lower levels of depression. For older adults, stable social interactions effectively alleviate loneliness and social isolation, thereby reducing the risk of depression (38). Strong-tie networks, such as neighborhood interactions and connections with family and friends, also serve as key channels for maintaining social engagement and enhancing life satisfaction (39). Therefore, in later life, when social networks tend to contract, BSC may serve as a potential mechanism linking the digital divide and depressive symptoms.

In summary, the three dimensions of digital divide may be associated with depressive symptoms through BSC. Based on this reasoning, the following hypothesis is proposed.

Hypothesis 2:among The associations between the three dimensions of the digital divide and depressive symptoms among older adults may be partially mediated by bonding social capital (BSC).

## 3 Materials and methods

### 3.1 Data source

The data are drawn from the China Family Panel Studies (CFPS), a nationally representative longitudinal survey conducted by the Institute of Social Science Survey at Peking University (40). Initiated in 2010, CFPS adopts households as the primary tracking unit and covers 25 provinces (autonomous regions and municipalities) in China. The survey provides systematic measurements of Internet use behaviors. It includes not only basic information on whether individuals use the Internet, but also detailed items on types of online activities and evaluations of the importance of the Internet across different life domains. These can provide strong support for this study.

**Figure 1:**
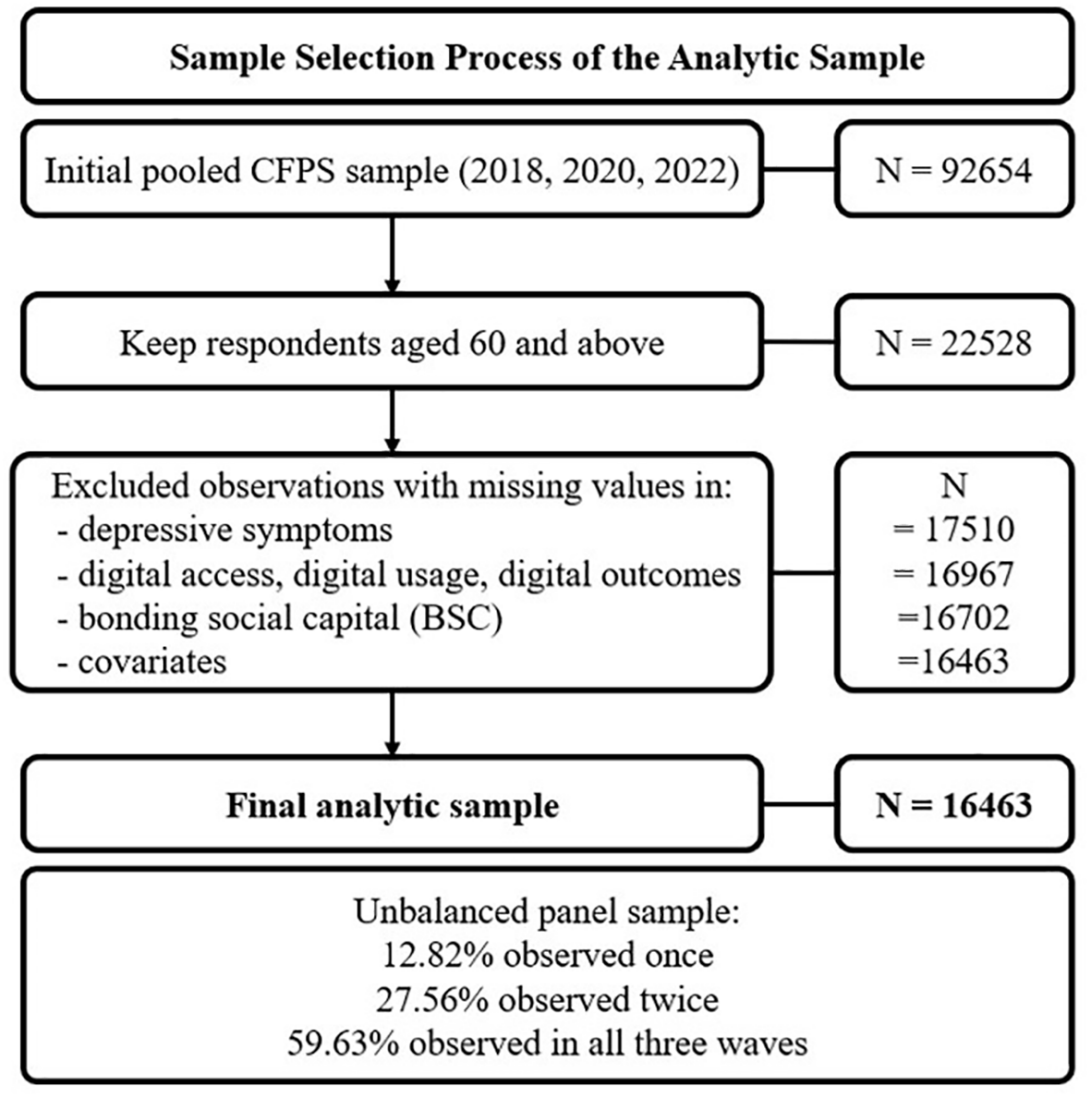
Sample selection process of the analytic sample.

Among This study utilizes data from the 2018, 2020, and 2022 waves of the survey to construct an unbalanced panel dataset and conducts the statistical analyses using Stata 18.0. The selection of these three waves was based on two main considerations. First, Internet penetration in China continued to increase during this period, providing an important observational window for examining the digital divide among older adults. Second, relatively complete and comprehensive questionnaire items were available for constructing the core variables of this study. In terms of sample selection, the study population was restricted to respondents aged 60 years and above. The sample selection process is presented in **Figure 1**, resulting in a final sample of 16,463 valid observations. Prior to model estimation, diagnostic tests were conducted for the main variables, and the results indicated no serious multicollinearity among the variables.

### 3.2 Variables

#### 3.2.1 Dependent variable

Depressive symptoms are measured using eight items from the CFPS questionnaire on the frequency of emotions and behaviors experienced over the past week. These items are derived from the short version of the Center for Epidemiologic Studies Depression Scale (CES-D) (41), which effectively captures short-term depressive symptomatology. The Cronbach’s α is approximately 0.781, indicating acceptable reliability. Each item is scored from 1 to 4, and the summed score forms a continuous index of depressive symptoms. Higher values indicate more severe depressive symptoms.

#### 3.2.2 Independent variables

In digital divide research, Internet access is typically regarded as the most fundamental form of digital inequality. It reflects whether individuals possess the basic conditions necessary to access digital resources. Accordingly, at the level of digital access, this study constructs a binary variable based on CFPS questionnaire items asking whether respondents accessed the Internet through mobile devices or computers. A value of 1 indicates that the individual has achieved digital access, whereas a value of 0 indicates no digital access.

At the level of digital usage, the literature further distinguishes between different types of online activities. Usage gap theory classifies Internet use into entertainment-oriented activities and functional uses oriented toward information acquisition, learning, or social interaction (17, 42). Based on the functional attributes of digital usage, this study classifies digital usage into entertainment-oriented usage, instrument-oriented usage, and mixed usage. Entertainment-oriented usage mainly refers to Internet activities primarily intended for leisure and entertainment, including online gaming and short-video viewing. Instrument-oriented usage mainly refers to Internet activities aimed at information acquisition, learning, or task processing, including online transactions, online learning, and work-related activities. Considering the differences in Internet activity items across different waves of the CFPS survey, this study prioritizes Internet activity indicators with relatively high comparability across survey years and relatively clear functional attributes. Specifically, the commercial activity items in the 2018 questionnaire (e.g., online banking and online shopping) were matched with the online shopping items in the 2020 and 2022 waves. The entertainment items in the 2018 questionnaire (e.g., watching videos and downloading songs) mainly corresponded to short-video viewing in later waves. In addition, given the importance of online social interaction in the digital lives of older adults, this study incorporates indicators of online social interaction, including “using the Internet for social activities (e.g., chatting and posting on Weibo)” in the 2018 questionnaire and “frequency of posting updates about work and daily life on social media” in the 2020 and 2022 questionnaires. These activities simultaneously involve entertainment, social interaction, information acquisition, and daily-life services, thereby exhibiting strong hybrid characteristics that make them difficult to classify into a single usage category. Therefore, this study further constructs a mixed usage variable, referring to the simultaneous engagement in entertainment-oriented, instrument-oriented, and social usage, in order to capture deeper levels of digital participation and digital embeddedness among older adults.

In addition, digital divide research increasingly focuses on disparities in the outcomes of Internet use. Because such outcomes are difficult to measure directly, this study uses individuals’ subjective evaluations of the value of the Internet as a proxy variable for digital outcomes. In general, a higher perceived importance of the Internet in specific life domains indicates greater reliance and, by implication, greater access to information, resources, or convenience. Following this approach, the digital outcomes indicator was constructed based on respondents’ evaluations of the importance of the Internet in areas including work, entertainment, social connection, learning, and daily-life affairs (**Supplementary Table 1**). The Cronbach’s α coefficient for these indicators was 0.802, indicating high internal consistency. Further KMO and Bartlett’s tests were conducted, yielding a KMO value of 0.820 and a significant Bartlett’s test of sphericity (p < 0.001), suggesting strong correlations among the variables. Subsequently, principal component analysis (PCA) was employed to extract the first principal component for constructing the digital outcomes index. The results showed that the eigenvalue of the first principal component was 2.80, explaining 56% of the total variance. All indicators exhibited relatively high loadings on the first principal component, indicating strong consistency in perceived Internet importance across different domains of daily life. Therefore, the factor scores of the first principal component were ultimately used as the indicator of digital outcomes to measure perceived digital outcomes at the individual level.

To further examine the validity of this proxy variable, this study assessed the associations between the digital outcomes index and several objective socioeconomic indicators (**Supplementary Table 2**). The results showed that the digital outcomes index was significantly positively associated with income level and exhibited higher levels among employed individuals. These findings suggest that the digital outcomes index can, to some extent, reflect individuals’ digital outcomes.

#### 3.2.3 Mediating variable

Putnam (31) classified social capital into bridging social capital and bonding social capital. In the operationalization of variables, two considerations are relevant. First, within older adults’ social networks, family and neighborhood ties constitute the primary sources of social capital, whereas BrSC derived from cross-group interactions is relatively limited. Second, due to constraints in the CFPS questionnaire, BrSC cannot be systematically measured. Therefore, this study focuses on BSC. An index system is constructed from five dimensions: family caregiving support (household chores and daily-life care), parent–child relationship quality, frequency of face-to-face interactions, frequency of contact (via telephone calls, text messages, letters, and emails), and neighborhood trust. For items related to multiple children, row means are calculated to obtain aggregate measures of support and interaction. All variables are then standardized and averaged to construct a composite index of BSC. Higher values indicate higher levels of BSC.

#### 3.2.4 Control variables

Control variables are selected based on the health capital model and well-established determinants in prior research on older adults’ health (8, 43). Demographic controls include age and gender. Given the importance of education and family structure for social support, additional controls include education, marital status, and number of children (2, 44). Residence and region are also included to account for regional disparities in resource allocation. Health status is captured by self-rated health to control for baseline physical health effects on depressive symptoms. Participation in basic social medical insurance is included to reflect institutional support conditions (45). Descriptive statistics for the main variables are reported in **Table 1**.

**Table 1:** Descriptive statistics of main variables (N = 16,463).

Variable	Mean	SD	Min	Max
Depressive symptoms	13.741	5.411	8	32
Digital access	0.513	0.500	0	1
Digital usage	0.605	1.190	0	4
Digital outcome	0.009	0.788	-2.127	1.056
BSC	-0.004	0.592	-3.194	1.688
Gender	0.516	0.499	0	1
Age	68.030	5.802	60	104
Marital status	0.825	0.380	0	1
Number of children	2.132	1.219	0	8
Education	5.555	4.646	0	19
Residence	0.313	0.464	0	1
Region	1.791	0.811	1	3
Self-rated health	2.577	1.246	1	5
Medical insurance	0.920	0.272	0	1

### 3.3 Analytical strategy

The empirical analysis proceeds in four steps. check First, this study employs a two-way fixed effects model. In addition to controlling for individual fixed effects, year fixed effects are further incorporated to account for time-invariant individual heterogeneity and the common influence of changes in the broader social environment. The effects of the digital divide on depressive symptoms among older adults in China are examined from the three dimensions of digital access, digital usage, and digital outcomes. Cluster-robust standard errors are used in the model estimation, with clustering at the individual level, to mitigate heteroskedasticity and serial correlation in panel data. Second, robustness tests are conducted using propensity score matching (PSM), sample restriction adjustments, and replacement of the dependent variable. Third, the Bootstrap method is employed to test the mediating effect of BSC. Finally, period-based heterogeneity analyses are conducted, and a Chow-type structural breakpoint test is applied to examine whether the COVID-19 pandemic altered the association structure between the digital divide and depressive symptoms.

## 4 Results

### 4.1 The three dimensions of the digital divide and depressive symptoms among older adults

To examine the association between Internet access and depressive symptoms among older adults, this study employs a two-way fixed effects model while controlling for both individual and year fixed effects, thereby minimizing potential bias arising from unobserved individual heterogeneity and temporal changes. The results are presented in Model 1 (M1) of **Table 2**. The findings indicate that Internet access is significantly negatively associated with depressive symptoms among older adults (p < 0.05). Specifically, compared with periods without Internet access, older adults tend to exhibit lower levels of depressive symptoms after gaining access to the Internet. Therefore, Hypothesis 1a is supported.

**Table 2:** Effects of digital divide on depressive symptoms among older adults.

Variable	M1	M2	M3
Digital access	-0.219* (0.106)		
Entertainment use		-0.208* (0.104)	
Instrumental use		-0.235* (0.102)	
Mixed use		-0.135* (0.058)	
Digital outcomes			-0.448** (0.163)
Year (reference group: 2018)			
2020	-1.074*** (0.322)	-0.885** (0.317)	-1.441*** (0.359)
2022	-1.828** (0.621)	-1.633** (0.622)	-2.507*** (0.531)
Control variable	Yes	Yes	Yes
*N*	16463	16463	4665
Within R²	0.0325	0.0326	0.0362

*p < 0.05, **p < 0.01, ***p < 0.001. The full model results (including control variables, F-statistic and 95% confidence intervals) are reported in the **Supplementary Table 3**.

Building on this analysis, different patterns of Internet usage are further distinguished, with the results reported in Model 2 (M2). Compared with non-Internet users, entertainment-oriented usage (p < 0.05), instrument-oriented usage (p < 0.05), and mixed usage (p < 0.05) are all significantly associated with lower levels of depressive symptoms. Thus, Hypothesis 1b is supported.

Model 3 (M3) presents the relationship between digital outcomes and depressive symptoms. The results show that digital outcomes are significantly negatively associated with depressive symptoms among older adults (p < 0.01). This suggests that older adults who perceive the Internet as more important and report greater subjective benefits from digital usage tend to exhibit relatively lower levels of depressive symptoms. Therefore, Hypothesis 1c is supported.

In addition, the coefficients of the year variables in **Table 2** indicate that, after controlling for individual fixed effects and related covariates, the overall levels of depressive symptoms in 2020 and 2022 were lower than those in 2018, with the absolute value of the coefficient for 2022 being larger than that for 2020. This finding suggests that depressive symptoms among older adults showed a declining trend during the study period, potentially due to improved digital adaptation, increased online entertainment and online social interaction, and the expansion of digital public services. Nevertheless, such changes may also reflect the combined influence of sample composition changes and period effects, and therefore should be interpreted with caution.

### 4.2 Robustness checks

To further validate the robustness of the baseline results, we conducted several tests, including propensity score matching (PSM), sample restriction, and alternative dependent variable specifications.

First, to address potential selection bias associated with Internet access, PSM is employed. As shown in **Table 3**,neighbor propensity scores were estimated based on a series of pre-treatment covariates, including age, gender, marital status, educational attainment, hukou status, region, physical health status, insurance coverage, and number of children. A 1:4 nearest-neighbor matching method with replacement was applied to construct the treatment and control groups. At the same time, the common support condition was imposed to exclude observations whose propensity scores fell outside the overlapping region. The sample sizes before and after matching were 8,210 and 8,251, respectively. Balance tests indicate substantial improvement after matching. The mean bias decreases from 29.7% before matching to 2.7% after matching. Rubin’s B falls to 12.4, well below the acceptable threshold of 25%. All covariate biases are reduced to below 10%. **Table 4** shows that, t-tests are no longer statistically significant. This confirms that the matched samples achieve satisfactory balance. The average treatment effect on the treated (ATT) estimated based on the matched samples remained statistically significant, and the direction of the results was consistent with those of the baseline models. In addition, this study further employed 1:1 nearest-neighbor matching and caliper matching (with a caliper width of 0.05), combined with a Bootstrap procedure based on 500 replications. The results, presented in **Table 5**, further confirm the robustness of the findings.

**Table 3:** Covariate balance test results of 1:4 nearest neighbor matching.

Variable	Sample	Mean (treated)	Mean (control)	% std. bias	P-value
Age	Unmatched	66.948	69.228	-40.0	0.000
	Matched	66.949	66.808	2.5	0.082
Gender	Unmatched	0.54606	0.47395	14.5	0.000
	Matched	0.54595	0.54415	0.4	0.811
Marital status	Unmatched	0.83762	0.80698	8.0	0.000
	Matched	0.83759	0.84491	-1.9	0.185
Education	Unmatched	6.0439	3.6742	44.4	0.000
	Matched	6.041	5.9245	2.2	0.143
Residence	Unmatched	0.39215	0.22291	37.3	0.000
	Matched	0.39201	0.38397	1.8	0.275
Central region	Unmatched	0.29752	0.29337	0.9	0.550
	Matched	0.29758	0.30671	-2.0	0.189
Western region	Unmatched	0.23309	0.26814	-8.1	0.000
	Matched	0.23315	0.23615	-0.7	0.639
Self-rated health	Unmatched	2.5969	2.5452	4.1	0.006
	Matched	2.5968	2.5714	2.0	0.173
Medical insurance	Unmatched	0.93237	0.8886	14.3	0.000
	Matched	0.93236	0.93673	-1.4	0.254
Number of children	Unmatched	2.0265	2.2198	-15.7	0.000
	Matched	2.0268	2.0482	-1.7	0.221

**Table 4:** Propensity score test to assess quality of matching.

Sample	Pseudo−R²	LR chi2	p>chi2	MeanBias	MedBias	Rubin’s B	Rubin’s R
Unmatched	0.076	1816.03	<0.001	18.7	14.4	66.6*	1.06
Matched	0.001	14.08	0.169	1.7	1.8	5.7	1.08

If B > 25%, R outside [0.5, 2].

**Table 5:** ATT estimates after propensity score matching.

Methods	ATT	SE	T-test	P-value	95% CI
1:4 nearest neighbor	-0.361	0.087	4.15	<0.001	(-0.535, -0.187)
1:1 nearest neighbor	-0.371	0.110	-3.37	<0.001	(-0.585, -0.157)
Caliper matching	-0.371	0.110	-3.37	<0.001	(-0.575, -0.167)

The 95% confidence interval was obtained from 500 resamples using the bootstrap method.

Second, considering that individuals aged over 90 have relatively low rates of Internet access and use, and that the influence of the Internet on their lifestyles and social interactions may be limited, we restricted the sample to those aged 60–90. Re-estimation shows that the direction and significance of the core explanatory variables remain substantively unchanged.

Finally, the depressive symptoms measure was transformed into a binary variable, and a mixed-effects logit model was employed for analysis. The construction of the binary variable follows Radloff (46) and has been validated in Zhang & Liang (47), where the 80th percentile of the CES-D 8-item scale (i.e., a cutoff value of 17) is used as the threshold. Scores greater than or equal to 17 are classified as exhibiting depressive symptoms and coded as 1, while scores below 17 are considered normal and coded as 0. The rationale for using this model lies in the ability of the mixed-effects logit specification to incorporate individual-level random effects in panel data, thereby partially accounting for unobserved intra-individual dependence. Moreover, for a binary outcome variable, the results can capture the directional associations between different dimensions of the digital divide and the risk of depressive symptoms among older adults. The results are broadly consistent with those of the baseline fixed effects models (**Supplementary Table 5**), indicating the robustness of the main findings.

### 4.3 Explanatory pathways of BSC

To further explore the potential social pathways linking the three dimensions of the digital divide and depressive symptoms among older adults, this study employs the KHB method and Bootstrap procedures to examine the mediating role of BSC. The results are presented in **Table 6**.

**Table 6:** Indirect association decomposition through bonding social capital (BSC).

Variable	Total association	Direct association	Indirect association	Bootstrap CI	% associated
Digital access	0.523*** (0.067)	0.492*** (0.067)	0.031** (0.009)	(0.019, 0.060)	5.95%
Entertainment use	0.457*** (0.064)	0.406*** (0.064)	0.051*** (0.009)	(0.038, 0.076)	11.25%
Instrumental use	0.190*** (0.034)	0.160*** (0.034)	0.030*** (0.005)	(0.024, 0.043)	15.85%
Mixed use	0.149*** (0.025)	0.126*** (0.025)	0.023*** (0.004)	(0.019, 0.033)	15.40%
Digital outcomes	0.135! (0.079)	0.099 (0.068)	0.036** (0.014)	(0.010, 0.063)	26.67%

! p < 0.1, ** p < 0.01, *** p < 0.001. Bootstrap confidence intervals were estimated based on 1,000 resampling replications.

BSC exhibits significant indirect pathways in the associations between all dimensions of the digital divide and depressive symptoms among older adults. Specifically, in the relationship between Internet access and depressive symptoms, the mediating proportion of BSC is 5.95%. At the level of Internet usage, the mediating proportions are 11.25% for entertainment-oriented usage, 15.85% for instrument-oriented usage, and 15.40% for mixed usage. At the level of digital outcomes, the mediating proportion reaches 26.67%. Overall, as the digital divide progresses from access to usage and further to outcomes, the indirect role of BSC becomes increasingly pronounced. Social tie maintenance, strengthened emotional interaction, and enhanced access to social support formed through digital engagement among older adults may help explain the relationship between digitalization and mental health. Therefore, Hypothesis 2 is supported.

### 4.4 Period heterogeneity analysis

Considering that the COVID-19 pandemic broke out in late 2019 and early 2020, it may have altered individuals’ Internet use context, promoting the rapid expansion of online entertainment, online social interaction, and digital public services, thereby potentially affecting the associations between digital access, digital usage, digital outcomes, and depressive symptoms. Accordingly, this study further divides the sample into the pre-pandemic period (2018) and the pandemic period (2020 and 2022), and conducts subgroup regression analyses of the core explanatory variables. The results are presented in **Table 7**.

**Table 7:** Results of period heterogeneity analysis.

Variable	Pre-pandemic	Pandemic	Pre-pandemic	Pandemic	Pre-pandemic	Pandemic
Digital access	-0.445***	-0.640***				
	(0.127)	(0.101)				
Entertainment use			-2.173***	-3.727*		
			(0.391)	(1.597)		
Instrumental use			-1.079**	-0.599***		
			(0.335)	(0.166)		
Mixed use			-1.277***	-0.672***		
			(0.225)	(0.109)		
Digital outcomes					-0.320	-0.380*
					(0.229)	(0.187)
Control variable	Yes	Yes	Yes	Yes	Yes	Yes
*N*	7588	8875	6517	9946	353	4312
R²	0.1842	0.1550	0.1875	0.1552	0.2218	0.1797

* p < 0.05, ** p < 0.01, *** p < 0.001.

The subgroup regression results show that, in the pre-pandemic period, digital access (β = -0.445, p < 0.001), entertainment-oriented usage (β = -2.173, p < 0.001), instrument-oriented usage (β = -1.079, p < 0.01), and mixed usage (β = -1.277, p < 0.001) are all significantly negatively associated with depressive symptoms, whereas digital outcomes (β = -0.320) are not statistically significant. In the post-pandemic period, digital access remains significant (β = -0.640, p < 0.001), while entertainment-oriented usage (β = -3.727, p < 0.05), instrument-oriented usage (β = -0.599, p < 0.001), mixed usage (β = -0.672, p < 0.001), and digital outcomes (β = -0.380, p < 0.05) are all negatively associated with depressive symptoms. Overall, the associations between key digital variables and depressive symptoms among older adults remain stable before and after the pandemic.

To examine whether the pandemic induced a structural break, this study applies a Chow-type test to compare regression coefficients across the two periods. The results indicate that, except for mixed usage (p = 0.036), no significant structural breaks are observed for the other explanatory variables. Overall, the direction and magnitude of the effects of key digital variables remain broadly stable before and after the pandemic.

## 5 Discussion

This study focuses on the intersection of digital transformation and population aging in China. Using nationally representative survey data, it examines the associations between the digital divide and depressive symptoms among older adults in China, as well as the underlying social mechanisms. The findings indicate that the impact of digital technologies on depressive symptoms extends beyond a purely technical effect. Instead, it represents a complex process embedded within social network structures and broader societal transformations. Empirically, the study contributes to the literature on the digital divide and well-being in later life and offers a new perspective on pathways to active aging in the digital era in China.

### 5.1 The multidimensional association between the digital divide and depressive symptoms among older adults

At the level of digital access, digital access is significantly associated with lower levels of depressive symptoms among older adults. With the development of digital governance and digital services, digital technologies have evolved from emerging tools into foundational infrastructure for social functioning. Internet access no longer merely signifies technological availability; it also reflects the opportunity to integrate into digital society. For older adults, successful Internet access often implies greater convenience in daily life and expanded opportunities for social interaction, thereby potentially mitigating the risk of social isolation during role transitions.

At the level of digital usage, the results reveal heterogeneous associations between different usage patterns and depressive symptoms. This finding empirically supports the theoretical shift in digital divide research from access to usage (17). As digital infrastructure becomes more widespread, disparities in access diminish, while differences in usage patterns and structures become increasingly salient (6, 16). Toward The results further show that instrument-oriented Internet usage is more strongly associated with lower levels of depressive symptoms, whereas entertainment-oriented usage exhibits relatively limited or even unstable associations. This suggests that the psychological significance of digital technologies does not depend on whether they are used, but rather on how they are used. When Internet use is embedded in social interaction processes and linked to everyday life needs, it is more likely to be associated with access to social support and to generate stable psychological resources.

At the level of digital outcome, behavior the study also finds a significant association between digital outcomes and lower levels of depressive symptoms among older adults. From the perspective of digital inclusion, digitalization is not only a technological process, but also a process through which individuals transform digital technologies into resources and capabilities in everyday life. When older adults are able to use the Internet to meet daily needs, maintain family ties, and enhance social participation, their sense of identification with, control over, and efficacy in the digital environment may also increase, thereby being associated with more positive psychological states. This finding empirically supports the theoretical shift in digital divide research from an “access divide” to a “usage divide” and further to an “outcome divide” (Horrigan & Rainie, 2002; 13).

It should be noted that, although the effect sizes of the associations between digital divide-related variables and depressive symptoms are generally modest, they still carry practical significance among older adults. Depressive symptoms in later life are often jointly shaped by multiple factors, including life-course experiences, physical health, functional limitations, family support, social participation, and economic conditions. As a result, a single social factor is unlikely to produce substantial changes in psychological well-being. At the same time, the overall level of digital participation among older adults remains relatively limited, and considerable heterogeneity exists in digital skills and technological adaptability. In this context, even modest improvements in digital access or digital usage may signify enhanced maintenance of social connections, improved convenience in daily life, and expanded access to social support, thereby generating cumulative positive effects on psychological well-being. Therefore, although the effect sizes associated with digital divide-related variables appear relatively moderate, they still possess important implications for public health and social governance in the context of concurrent population aging and digitalization.

### 5.2 The role of BSC: social connections and psychological support

This study further finds that BSC exhibits significant indirect pathways in the association between the digital divide and depressive symptoms. Overall, as the digital divide extends from access to usage and further to outcomes, the mediating role of social capital becomes increasingly pronounced. This indicates that the relationship between digital technologies and mental health among older adults does not arise solely from the technology itself, but is also shaped through social mechanisms such as the maintenance of existing social relationships, the strengthening of emotional interaction, and the enhancement of perceived social support.

Socioemotional selectivity theory suggests that as individuals age, their perceived future time horizon shortens, and social goals increasingly prioritize immediate emotional satisfaction. Under this shift in temporal perception, older adults tend to selectively narrow their social networks and invest more in a limited number of emotionally meaningful relationships (32, 34). Consequently, support from family members and close neighbors becomes the primary resource for mitigating depression risk and maintaining psychological well-being. In this context, the role of the Internet in social networks may be more about maintenance rather than expansion. Such emotionally supportive and reciprocal ties embedded in strong-tie networks may be further associated with better mental health outcomes (35, 48). In addition, the indirect effect proportion is relatively small at the level of digital access, whereas it is substantially higher at the levels of digital usage and digital outcomes. This suggests that the association between digital technologies and psychological well-being becomes more pronounced when digital technologies are truly embedded in individuals’ everyday lives.

### 5.3 Digital adaptation in the context of macro-level social change

This study, combined with the period heterogeneity analysis, suggests that the association between the digital divide and depressive symptoms among older adults is embedded in a dynamic process of digital transformation and broader macro-level social change. Overall, both before and during the COVID-19 pandemic, digital access, digital usage, and digital outcomes remain significantly associated with depressive symptoms among older adults. This indicates that digital technologies have gradually become an important determinant of older adults’ daily lives, and their role has not fundamentally changed in response to major macro-level shocks.

At the same time, the year effects in the fixed effects models show that depressive symptoms in 2020 and 2022 are overall lower than in 2018, with a larger absolute coefficient in 2022. This suggests a general declining trend in depressive symptoms among older adults over the study period. A possible explanation is that, with the continued advancement of digital society construction, some older adults have gradually developed routine technological adaptation through long-term exposure to and use of the Internet. The expansion of online entertainment, online social interaction, and digital public services has helped alleviate social isolation and enhanced older adults’ capacity to access information and maintain social connections. In particular, between 2020 and 2022, the extent to which digital technologies were embedded in daily life increased substantially. This temporal variation may also be jointly influenced by changes in sample composition, improvements in the social environment, and broader period effects (49), and therefore should be interpreted with caution.

Further structural break tests indicate that mixed usage exhibits significant differences before and after the pandemic. This may be related to the comprehensive expansion of online life functions during the pandemic period (50). Compared with single-purpose entertainment or instrument-oriented usage, mixed usage simultaneously incorporates information acquisition, task management, and social interaction, making it more likely to help older adults maintain social connections and everyday life routines during special periods. This finding also reflects heterogeneity in digital adaptation among older adults. For those capable of integrating multiple digital functions, better psychological outcomes are more likely to be observed. Therefore, active aging in a digital context does not merely imply increasing Internet penetration; it also requires attention to the psychological resilience of older adults and the provision of greater social support, so as to mitigate inequality effects arising from technological change.

### 5.4 Limitations and future research

This study has several limitations that indicate directions for future research. among First, this study uses individuals’ evaluations of the importance of the Internet as a proxy for digital outcomes. Although this indicator to some extent captures individuals’ overall perceived value of the Internet and is correlated with objective indicators such as income and employment status, it remains fundamentally a subjective measure and may be subject to psychological attribution bias and social desirability bias. Future research could incorporate objective indicators for measurement. Second, fixed effects models cannot rule out all potential confounding from unobserved factors, such as functional limitations and chronic disease history. Due to data limitations, this study is unable to fully control for all potential factors; therefore, the findings should not be interpreted as strict causal inference. Third, missing data among older adults may be non-random in nature. Individuals with poorer health status, lower digital skills, or more severe depressive symptoms may be more likely to have missing information or to drop out of the survey, making it difficult to fully rule out potential sample selection bias. Fourth, due to data constraints, this study cannot disentangle the effects of the COVID-19 pandemic from broader cohort effects or long-term digitalization trends. Future research could use additional waves of data or apply APC (Age–Period–Cohort) models to further distinguish these mechanisms. Fifth, because the available data do not provide sufficiently comparable indicators, this study is unable to adequately capture bridging social capital. This limitation may introduce bias in the estimation of indirect pathways. Future studies should incorporate more comprehensive measures of social capital in order to compare the distinct roles of different forms of social capital. Finally, the mediation analysis in this study is based on cross-sectional observations within the same survey waves, making it difficult to fully rule out the possibility of reverse causality. Future research could employ longer-term longitudinal data or use lagged variables to construct counterfactual mediation models, thereby improving methodological rigor.

## 6 Conclusion

This study utilizes three waves of longitudinal data from the CFPS 2018, 2020, and 2022 surveys, and employs a two-way fixed effects model, Bootstrap analysis, and propensity score matching (PSM), among other robustness checks, to systematically examine the association between the digital divide and depressive symptoms among older adults in China, as well as its underlying social mechanisms. The results show that, after controlling for individual fixed effects and year fixed effects, digital access, digital usage, and digital outcomes are all significantly associated with lower levels of depressive symptoms among older adults. This association remains robust across multiple specifications. In addition, bonding social capital exhibits significant indirect pathways in the association between the digital divide and depressive symptoms. Overall, as the digital divide extends from access to usage and further to outcomes, the mediating role of social capital becomes increasingly pronounced. In the period heterogeneity analysis, the study further distinguishes between the pre-pandemic and pandemic periods. The results indicate that the direction of the associations between digital access, digital usage, digital outcomes, and depressive symptoms remains broadly consistent across periods; however, mixed usage exhibits more pronounced differences between the two periods.

These findings provide empirical support for addressing the dual challenges of population aging and digital transformation. From a policy perspective, there is a need to shift from mere technological access to substantive digital empowerment. This includes improving age-friendly digital services and content provision, as well as enhancing digital literacy and risk awareness among older adults. The goal is to build an inclusive digital society that combines efficiency with social sensitivity, ensuring that technological advancement aligns with human-centered care and ultimately promotes healthy aging in the digital era.

## Data Availability

Publicly available datasets were analyzed in this study. This data can be found here: https://cfpsdata.pku.edu.cn/#/home.
