# Supplementary material for: From digital access to social connectedness: the digital divide, bonding social capital, and depressive symptoms among older adults in China
Source: Front Psychiatry. 2026 Jun 16;17:1845066. doi: 10.3389/fpsyt.2026.1845066 (PMC13314774; doi:10.3389/fpsyt.2026.1845066)
Supplement: Supplementary file 1 [file Supplementaryfile1.docx]

Supplementary Material

# Supplementary Figures


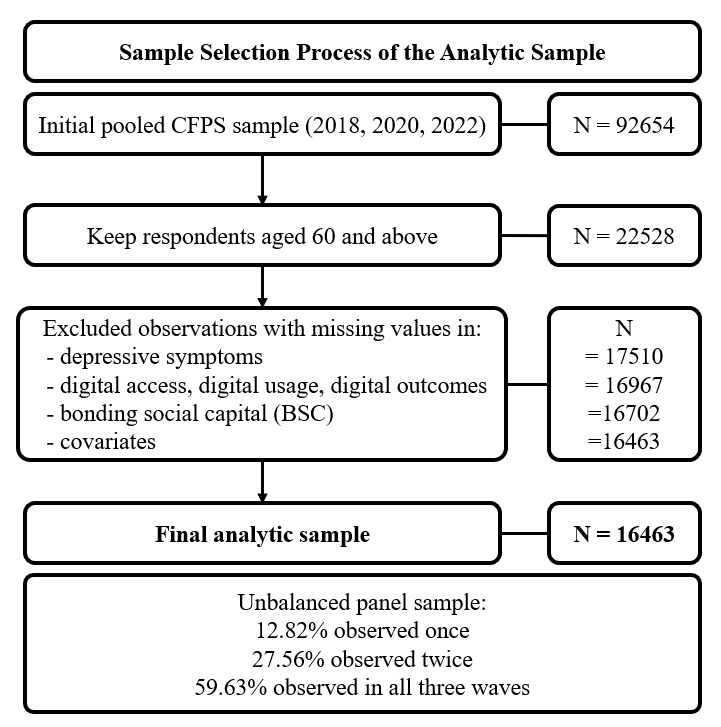


Figure 1. Sample Selection Process of the Analytic Sample

# Supplementary Tables

Table S1. PCA Results for Digital Outcomes Index

| Variables | Factor Loading |
| --- | --- |
| Internet importance in work | 0.6914 |
| Internet importance in entertainment | 0.6952 |
| Internet importance in social contact | 0.5727 |
| Internet importance in study | 0.7158 |
| Internet importance in daily life | 0.6230 |

| Statistics | Value |
| --- | --- |
| KMO | 0.8018 |
| Bartlett’s Test | P<0.001 |
| Eigenvalue of Comp1 | 2.8136 |
| Variance explained | 56.27% |

Table S2 Construct Validity Checks of Digital Outcomes Index

|  | Digital outcomes | Digital outcomes |
| --- | --- | --- |
| Employment | 0.177** |  |
|  | (0.0667) |  |
| Income |  | 0.0571* |
|  |  | (0.0245) |
| Age | -0.110 | -0.0895 |
|  | (0.116) | (0.117) |
| Agesq | 0.0527 | 0.0379 |
|  | (0.0849) | (0.0854) |
| Marital | 0.0482 | 0.0601 |
|  | (0.0857) | (0.0854) |
| Hukou | 0.252*** | 0.184** |
|  | (0.0644) | (0.0587) |
| Region (reference group: Eastern) |  |  |
| Central | 0.0677 | 0.0780 |
|  | (0.0652) | (0.0652) |
| Western | 0.181* | 0.209** |
|  | (0.0773) | (0.0773) |
| Insurance | 0.0149 | 0.0168 |
|  | (0.0855) | (0.0855) |
| _cons | 4.639 | 4.007 |
|  | (3.976) | (4.006) |
| N | 17203 | 17203 |
| R² | 0.0202 | 0.0198 |

Note: ^!^ *p* < 0.1, ^*^ *p* < 0.05, ^**^ *p* < 0.01, ^***^ *p* < 0.001

Table S3 Effects of digital divide on depressive symptoms among older adults

|  | M1 | | | M2 | | | | | M3 | |  |
| --- | --- | --- | --- | --- | --- | --- | --- | --- | --- | --- | --- |
|  | Coeff. (SE) | | Std β | | Coeff. (SE) | | Std β | | Coeff. (SE) | | Std β |
| Digital access | -0.219* (0.106) | -0.020 | |  | |  | |  | |  | |
|  | (-0.011, -0.427) |  | |  | |  | |  | |  | |
| Entertainment use |  |  | | -0.208* (0.104) | | -0.046 | |  | |  | |
|  |  |  | | (-0.003, -0.412) | |  | |  | |  | |
| Instrumental use |  |  | | -0.235* (0.102) | | -0.052 | |  | |  | |
|  |  |  | | (-0.036, -0.434) | |  | |  | |  | |
| Mixed use |  |  | | -0.135* (0.058) | | -0.030 | |  | |  | |
|  |  |  | | (-0.249, -0.021) | |  | |  | |  | |
| Digital outcomes |  |  | |  | |  | | -0.448** (0.163) | | -0.065 | |
|  |  |  | |  | |  | | (-0.128, -0.767) | |  | |
| Year(2018) |  |  | |  | |  | |  | |  | |
| 2020 | -1.074*** (0.322) |  | | -0.885** (0.317) | |  | | -1.441*** (0.359) | |  | |
|  | (-0.443, -1.704) |  | | (0.260, 1.507) | |  | | (-0.737, -2.145) | |  | |
| 2022 | -1.828** (0.621) |  | | -1.633** (0.622) | |  | | -2.507*** (0.531) | |  | |
|  | (-0.610, -3.046) |  | | (-0.413, -2.857) | |  | | (-1.466, -3.548) | |  | |
| Age | -0.287 (0.309) |  | | -0.349 (0.310) | |  | | -0.316 (0.764) | |  | |
|  | (-0.893, 0.319) |  | | (-0.952, 0.264) | |  | | (-1.814, 1.181) | |  | |
| Age square | -0.201 (0.197) |  | | -0.155 (0.198) | |  | | -0.310 (0.543) | |  | |
|  | (-0.587, 0.186) |  | | (-0.546, 0.228) | |  | | (-1.375, 0.755) | |  | |
| Marital | 1.349*** (0.328) |  | | 1.345*** (0.328) | |  | | 1.362 (1.240) | |  | |
|  | (0.707, 1.991) |  | | (0.700, 1.986) | |  | | (-1.069, 3.794) | |  | |
| Hukou | -0.283 (0.244) |  | | -0.279 (0.244) | |  | | 0.108 (0.487) | |  | |
|  | (-0.762, 0.196) |  | | (-0.756, 0.201) | |  | | (-0.846, 1.063) | |  | |
| Region (Eastern) |  |  | |  | |  | |  | |  | |
| Central | -0.300 (0.629) |  | | -0.306 (0.632) | |  | | -0.023 (0.879) | |  | |
|  | (-1.533, 0.933) |  | | (-1.560, 0.921) | |  | | (-1.746, 1.701) | |  | |
| Western | -0.646 (0.961) |  | | -0.647 (0.963) | |  | | -2.019 (1.442) | |  | |
|  | (-2.529, 1.237) |  | | (-2.526, 1.246) | |  | | (-4.847, 0.809) | |  | |
| Physical health | -0.479*** (0.048) |  | | -0.479*** (0.048) | |  | | -0.409** (0.127) | |  | |
|  | (-0.384, -0.574) |  | | (-0.384, -0.573) | |  | | (-0.159, -0.658) | |  | |
| Insurance | -0.499* (0.238) |  | | -0.500* (0.238) | |  | | 0.135 (0.393) | |  | |
|  | (-0.032, -0.966) |  | | (-0.032, -0.967) | |  | | (-0.635, 0.905) | |  | |
| Status | -0.154*** (0.044) |  | | -0.154*** (0.044) | |  | | 0.002 (0.131) | |  | |
|  | (-0.067, -0.240) |  | | (-0.067, -0.240) | |  | | (-0.254, 0.258) | |  | |
| Number of children | 0.089 (0.103) |  | | 0.089 (0.103) | |  | | 0.088 (0.318) | |  | |
|  | (-0.113, 0.292) |  | | (-0.113, 0.292) | |  | | (-0.536, 0.711) | |  | |
| F-statistic | 15.80*** |  | | 16.16*** | |  | | 8.03*** | |  | |
| N | 17150 |  | | 17150 | |  | | 3665 | |  | |
| Within R² | 0.0325 |  | | 0.0326 | |  | | 0.0362 | |  | |

Note: ! p < 0.1, * p < 0.05, ** p < 0.01, *** p < 0.001. The row immediately following each variable's coefficient represents the 95% confidence interval (CI).

Table S4 Characteristics of Included and Excluded Observations

| Variables | Included | Excluded | p-value |
| --- | --- | --- | --- |
| Digital access | 0.79 (0.41) | 0.27 (1.22) | <0.001 |
| Digital usage | 1.92 (1.30) | 0.01 (0.12) | <0.001 |
| Digital outcomes | 0.01 (0.79) | 0.31 (0.95) | 0.023 |
| Depressive symptom | -26.93 (4.25) | -18.81 (12.24) | <0.001 |
| Age | 66.30 (5.07) | 69.90 (6.52) | <0.001 |
| Gender | 0.56 (0.50) | 0.48 (0.50) | 0.247 |
| Marital | 0.87 (0.33) | 0.81 (0.40) | <0.001 |
| Hukou | 0.52 (0.50) | 0.25 (0.43) | <0.001 |
| Region | 1.72 (0.78) | 1.86 (0.83) | <0.001 |
| Physical health | 2.74 (1.17) | 2.42 (1.25) | <0.001 |
| Insurance | 0.92 (0.27) | 0.88 (0.38) | <0.001 |
| Status | 3.31 (1.05) | 3.47 (1.15) | <0.001 |
| Number of children | 1.78 (1.02) | 1.63 (1.46) | <0.001 |

Note: Values are presented as means with standard deviations in parentheses for continuous variables, and proportions for categorical variables. P-values are based on two-sample t-tests for continuous variables and chi-square tests for categorical variables. Included observations refer to observations retained in the final analytic sample after listwise deletion, whereas excluded observations refer to observations removed due to missing values in key variables.

Table S5 Mixed-effects logistic regression of binary depressive symptom on digital divide (Robustness Check)

|  | M1 | M2 | M3 |
| --- | --- | --- | --- |
| Digital access | -0.231^***^ |  |  |
|  | (0.0637) |  |  |
| Entertainment use |  | -1.139^*^ |  |
|  |  | (0.497) |  |
| Instrument use |  | -0.191 |  |
|  |  | (0.258) |  |
| Mixed use |  | -0.387^***^ |  |
|  |  | (0.0793) |  |
| Digital outcomes |  |  | -0.195^*^ |
|  |  |  | (0.0846) |
| Year (2018=0) |  |  |  |
| 2020 | -0.0965 | 0.0717 | 0.0343 |
|  | (0.0681) | (0.0576) | (0.296) |
| 2022 | 0.133^*^ | 0.317^***^ | 0.264 |
|  | (0.0655) | (0.0611) | (0.288) |
| age | -0.0375 | -0.0460 | 0.170 |
|  | (0.0963) | (0.0963) | (0.291) |
| Agesquare | 0.0126 | 0.0183 | -0.142 |
|  | (0.0688) | (0.0688) | (0.213) |
| Gender | -0.531^***^ | -0.529^***^ | -0.686^***^ |
|  | (0.0595) | (0.0593) | (0.148) |
| Marital | -0.860^***^ | -0.854^***^ | -1.277^***^ |
|  | (0.0743) | (0.0742) | (0.210) |
| Hukou | -0.837^***^ | -0.788^***^ | -1.365^***^ |
|  | (0.0695) | (0.0703) | (0.176) |
| Region (eastern=0) |  |  |  |
| Central | 0.191^**^ | 0.184^**^ | 0.485^**^ |
|  | (0.0698) | (0.0697) | (0.164) |
| Western | 0.619^***^ | 0.594^***^ | 1.089^***^ |
|  | (0.0717) | (0.0716) | (0.193) |
| Physical health | -0.621^***^ | -0.621^***^ | -0.694^***^ |
|  | (0.0241) | (0.0241) | (0.0702) |
| Insurance | -0.272^**^ | -0.269^**^ | 0.0999 |
|  | (0.0837) | (0.0837) | (0.247) |
| Status | -0.160^***^ | -0.168^***^ | -0.186^**^ |
|  | (0.0230) | (0.0230) | (0.0637) |
| Number of children | 0.0304 | 0.0255 | -0.134^!^ |
|  | (0.0237) | (0.0236) | (0.0715) |
| _cons | 3.667 | 3.874 | -3.201 |
|  | (3.352) | (3.352) | (9.884) |
| var(_cons[pid]) | 2.427^***^ | 2.394^***^ | 3.374^***^ |
|  | (0.189) | (0.188) | (0.848) |
| *N* | 17150 | 17150 | 3665 |
| Log_likelihood | -8333.387 | -8284.03 | -1524.6557 |

Note:! p < 0.1, * p < 0.05, ** p < 0.01, *** p < 0.001

Table S6 Baseline Comparison Between Completers and Dropouts

| Variable | Completers | Dropouts | p-value |
| --- | --- | --- | --- |
| Depression symptoms | 14.01 | 13.52 | <0.001 |
| Digital access | 0.76 | 0.82 | <0.001 |
| Digital usage | 0.26 | 0.37 | <0.001 |
| Digital outcomes | -0.14 | 0.09 | 0.0150 |
| Age | 68.46 | 67.28 | <0.001 |
| Gender | 0.51 | 0.50 | 0.730 |
| Marital | 0.79 | 0.84 | <0.001 |
| Hukou | 0.31 | 0.29 | 0.1414 |
| Region | 1.79 | 1.79 | 0.8270 |
| Physical health | 2.45 | 2.56 | 0.001 |
| Medical insurance | 0.92 | 0.94 | 0.0001 |
| Status | 3.37 | 3.42 | 0.0547 |
| Number of children | 2.24 | 2.18 | 0.0229 |
